# Parity and time-reversal violating nuclear forces with explicit $\Delta$-excitations

**DOI:** 10.1140/epja/s10050-024-01426-z

**Published:** 2024-10-28

**Authors:** L. Gandor, H. Krebs, E. Epelbaum

**Affiliations:** Institut für Theoretische Physik II, Ruhr-Universität Bochum, 44780 Bochum, Germany

## Abstract

We emphasize the usefulness of treating delta resonances as explicit degrees of freedom in applications of chiral effective field theory (EFT) to parity-violating and time-reversal-violating (PVTV) nuclear interactions. Compared with the delta-less framework, the explicit inclusion of the delta isobar allows one to resum certain types of contributions to the PVTV two-pion exchange two- and three-nucleon potentials without at the same time introducing any unknown parameters up to next-to-next-to-leading order in the EFT expansion. We provide the corresponding expressions for the delta contributions in momentum and coordinate spaces and compare the convergence of the EFT expansion in both formulations.

## Introduction

Parity-violating time-reversal-violating nuclear interactions play an important role in research focused on understanding the observed matter–antimatter asymmetry in the Universe and in searches for physics beyond the Standard Model. This includes, in particular, ongoing efforts towards an experimental observation of the permanent electric dipole moment of the nucleon and nuclei, which can only emerge from PVTV interactions. The CP violation in the Standard Model, which originates from complex phases in the quark and neutrino mixing matrices as well as from the $\theta$-term in the strong sector, is known to be insufficient to describe the observed matter–antimatter asymmetry. On the other hand, when viewing the Standard Model as a low-energy approximation in the EFT framework, further sources of CP violation appear from dimension-six and higher operators. In fact, many beyond-Standard-Model extensions including, e.g., supersymmetric models give rise to PVTV mechanisms, which are parametrized by higher-dimensional operators in the Standard Model EFT.

Regardless of the microscopic origin of CP violation in or beyond the Standard Model, a theoretical analysis of PVTV observables in nuclear systems can be most efficiently carried out in the framework of chiral EFT, which is tailored to describe phenomena at momentum scales relevant for nuclear physics. Here, the starting point is the most general effective Lagrangian written in terms of the relevant hadronic degrees of freedom, which respects the symmetries of the Standard Model (or Standard Model EFT), such as especially the spontaneously broken approximate chiral symmetry of QCD. CP violation can be incorporated in the effective chiral Lagrangian by introducing the corresponding terms, whose coupling constants are ultimately driven by microscopic mechanisms of CP violation and thus expected to be very small. The dominant CP violating interactions between pions and nucleons are parametrized in terms of four dimensionless low-energy constants (LECs) [1–4], which will be specified in the next section. The corresponding derivative-less vertices in the effective chiral Lagrangian are sufficient to determine the long-range PVTV nuclear interactions up to next-to-next-to-leading order (N$^2$LO) in the chiral EFT expansion. Microscopic mechanisms of CP violation lead to different scaling of these four LECs. Thus, by tuning these LECs to hypothetical experimental PVTV signals in nuclear systems one may be able to draw conclusions about specific sources of CP violation in or beyond the Standard Model.

PVTV nuclear forces have already been derived from the effective chiral Lagrangian up through N$^2$LO [3, 5, 6] and applied to study selected PVTV nuclear processes [3, 7]. PVTV nuclear current operators have been discussed in Refs. [6, 8]. For reviews of PVTV nuclear forces and currents and applications to nuclear systems see Refs. [9, 10]. However, all these studies are based on the formulation of chiral EFT in terms of pions and nucleons as the only degrees of freedom in the effective chiral Lagrangian. On the other hand, it is well known from studies of the usual parity-conserving time-reversal-conserving (PCTC) nuclear interactions that such a framework may suffer from convergence issues at low orders in the EFT expansion due to the implicit treatment of the $\Delta$(1232) resonance [11–16], which leads to large numerical values of some of the subleading pion-nucleon LECs. In this paper, we work out the long-range contributions to the nuclear forces mediated by intermediate delta-excitations up to N$^2$LO using the formulation of chiral EFT with explicit delta-isobar degrees of freedom. As discussed in the next section, the explicit inclusion of the delta-isobars does not lead to additional PVTV LECs, thereby allowing for a resummation of dominant higher-order contributions in a parameter-free fashion.

Our paper is organized as follows. In Sect. 2, we specify the effective Lagrangian relevant for our calculations, discuss the power counting and provide expressions for the renormalized two- (2N) and three-nucleon (3N) potentials up to N$^2$LO. The coordinate-space expressions for various long-range contributions are given in Sect. 3, where we also compare the convergence of the delta-less and delta-full formulations of chiral EFT. The main results of our study are briefly summarized in Sect. 4.

## PVTV potentials in momentum space

Both parity conserving and PVTV contributions to the effective chiral Lagrangian are well documented in the literature, see Refs. [17–20] and [1, 4], respectively. We therefore only list below terms relevant for our calculation, which emerge from expanding the covariantly transforming building blocks of the effective Lagrangian in powers of the pion fields:

1 $$\begin{aligned}  &   \mathcal {L}_{\pi \pi }^{\Delta _i=0} = \frac{1}{2} \partial _\mu {\varvec{\pi }}\cdot \partial ^\mu {\varvec{\pi }}- \frac{1}{2} M^2 {\varvec{\pi }}^2 + \ldots ,\nonumber \\  &   \mathcal {L}_{\pi N}^{\Delta _i=0} = N^\dagger \bigg [ i v \cdot \partial - \frac{1}{4 F^2} {\varvec{\tau }}\times {\varvec{\pi }}\cdot (v \cdot \partial {\varvec{\pi }}) - \frac{\mathring{g}_A}{F} {\varvec{\tau }}\cdot (S \cdot \partial {\varvec{\pi }}) \bigg ] N \nonumber \\  &   \qquad \qquad + \ldots , \nonumber \nonumber \\  &   \mathcal {L}_{\pi N}^{\Delta _i=1} = N^\dagger \bigg [- \frac{2 c_1}{F^2} M^2 {\varvec{\pi }}^2 + \frac{c_2}{F^2} ( v \cdot \partial {\varvec{\pi }}) \cdot (v \cdot \partial {\varvec{\pi }}) \nonumber \\  &   \qquad \qquad + \frac{c_3}{F^2} (\partial _\mu {\varvec{\pi }}) \cdot (\partial ^\mu {\varvec{\pi }}) - \frac{ic_4}{F^2} \Big [ S_\mu , \; S_\nu \Big ] {\varvec{\tau }}\times (\partial ^\nu {\varvec{\pi }}) \cdot (\partial ^\mu {\varvec{\pi }}) \bigg ] N \nonumber \\  &   \qquad \qquad + \ldots , \nonumber \nonumber \\  &   \mathcal {L}_{\pi N\Delta }^{\Delta _i=0} = - \frac{\mathring{h}_A}{F}N^\dagger {\varvec{T}}_\mu \cdot \partial ^\mu {\varvec{\pi }}+ \mathrm{H.c.} + \ldots , \nonumber \\  &   \mathcal {L}_{\pi \pi , \; \textrm{PVTV}}^{\Delta _i=-2} = \Delta _3 m_N \pi _3 {\varvec{\pi }}^2 + \ldots ,\nonumber \\  &   \mathcal {L}_{\pi N, \; \textrm{PVTV}}^{\Delta _i=-1} = N^\dagger \big [ g_0 {\varvec{\tau }}\cdot {\varvec{\pi }}+ g_1 \pi _3 + g_2 \pi _3 \tau _3 \big ] N + \ldots , \end{aligned}$$

where the ellipses refer to terms involving a larger number of pion fields ${\varvec{\pi }}$, which are not relevant for this work. Here, *N* and $\textrm{T}_\mu$ denote the large components of the nucleon and the $\Delta$ field in the Rarita–Schwinger formalism, respectively, which depend on the four-velocity *v*. Next, ${\varvec{\tau }}$ denote the isospin Pauli matrices, $S_\mu = -1/4 \gamma _5 [\gamma _\mu , \gamma _\nu ] v^\nu$ is the covariant spin operator of the nucleon, *M* is the pion mass to leading order in quark masses while *F*, $\mathring{g}_A$ and $\mathring{h}_A$ are the chiral-limit values of the pion decay constant, the nucleon and $\pi N \Delta$ axial couplings, respectively. Further, $c_i$, $i=1, \ldots , 4$ are LECs accompanying the subleading pion-nucleon vertices, while $\Delta _3$ and $g_i$ with $i=0, 1, 2$ are the LECs of the lowest-order pionic and pion-nucleon PVTV vertices. The appearance of the nucleon mass $m_N$ in $\mathcal {L}_{\pi \pi , \; \textrm{PVTV}}^{\Delta _i=-2}$ is just a matter of convention for keeping the PVTV LEC $\Delta _3$ dimensionless.

Notice that the PVTV effective chiral Lagrangian is obtained in the framework of the Standard Model EFT by integrating out high-energy scales above the hadronic one of the order of $\sim 1$ GeV [1–4]. This way, the LECs of the PVTV vertices of the effective chiral Lagrangian acquire additional dimensionless suppression factors, which depend upon specific microscopic scenarios of CP violation and are expressed in terms of the chiral symmetry breaking scale, Higgs vacuum expectation value, pion mass and decay constant and the relative difference of the up and down quark masses. For example, the CP violating three-pion coupling constant $\Delta _3$ appears to be relatively suppressed for most sources of CP violation. For a summary of the scaling of the LECs $\Delta _3$ and $g_i$ for various microscopic CP violation scenarios see the review article [10]. Throughout our paper, we use the same terminology as in Ref. [10] and treat all dimensionless PVTV LECs as quantities on equal footing of order $\mathcal {O} (1)$ for the sake of generality, i.e., we do not take into account suppression factors that may arise in specific microscopic CP violating scenarios unless explicitly stated otherwise. Clearly, the corresponding suppression factors must be included when studying phenomenological implications of various CP violating mechanisms on nuclear observables.

The superscripts of the effective Lagrangians in Eq. (1) refer to the vertex dimension $\Delta _i$ introduced in Ref. [21] and defined as

2 $$\begin{aligned} \Delta _i = \frac{n_i}{2} + d_i - 2\,, \end{aligned}$$

where $n_i$ is the number of baryon field operators and $d_i$ is the number of derivatives or pion mass insertion at a vertex of type *i*. The EFT order $Q^\nu$ of a connected *N*-nucleon irreducible^1^ diagram with *L* loops made out of $V_i$ vertices of type *i* is then given by [21, 22]

3 $$\begin{aligned} \nu = -4 + 2 (N+L) + \sum _i V_i \Delta _i \,. \end{aligned}$$

The expansion parameter of chiral EFT is given by $Q \in \{| \textbf{p} \, |/\Lambda _b, M_\pi /\Lambda _b \}$, with $M_\pi$ referring to the pion mass, $| \textbf{p} \, |\sim M_\pi$ to generic momenta of the nucleon and $\Lambda _b$ to the breakdown scale of the chiral EFT expansion. Throughout this work, we employ our usual counting for the nucleon mass with $m_N \sim \mathcal {O} (\Lambda _b^2/M_\pi ) \gg \Lambda _b$ [21, 23], which is different from the assignment $m_N \sim \mathcal {O} (\Lambda _b )$ adopted in Ref. [7]. Notice that we treat the LECs associated with leading-order (LO) three-pion vertex as $\Delta _3 m_N \sim \mathcal {O} (1)$.^2^

While parity-violating time-reversal-conserving interactions have already been considered in chiral EFT with explicit delta degrees of freedom [24, 25], PVTV nuclear forces from intermediate $\Delta$ excitations have, to the best of our knowledge, not been worked out yet at N$^2$LO. See, however, Ref. [26] for a discussion of the $\Delta$ contributions in the context of the QCD $\theta$-term.

However, it is easy to see that the lowest possible PVTV $\pi N \Delta$ terms must include at least one derivative in order to maintain Lorentz invariance, see also Ref. [26] for a related discussion. In the Rarita–Schwinger formulation, the $\Delta$ field appears in combination with the spin-3/2 projection operator

4 $$\begin{aligned} P_{\mu \nu } = g_{\mu \nu } - v_\mu v_\nu - \frac{4}{1-d} S_\mu S_\nu \,, \end{aligned}$$

where $g_{\mu \nu }$ is the Minkowski metric tensor and *d* the number of space-time dimensions. The relationships $v^2 = 1$, $v \cdot S = 0$ and $S^2 = (1-d)/4$ imply that both possible structures $N^\dagger v^\mu P_{\mu \nu } {\varvec{T}}^\nu$ and $N^\dagger S^\mu P_{\mu \nu } {\varvec{T}}^\nu$ vanish, and the only way to obtain a nonvanishing Lorentz-invariant $\pi N \Delta$ term is by contracting $P_{\mu \nu } {\varvec{T}}^\nu$ with a four-derivative. The argument becomes even more transparent if the Lagrangian is written directly in terms of a four-component spin-3/2 field as done e.g. in Ref. [12]. This requires the introduction of the $2 \times 4$ spin transition matrix $\textbf{S}$, which has to be contracted with a spatial derivative in order to restore rotational invariance. Thus, the lowest-order PVTV $\pi N \Delta$ vertices have $\Delta _i = 0$ and start contributing to the nuclear forces at next-to-next-to-next-to-leading order $Q^2$ (N$^3$LO), which is beyond the accuracy level of our calculation.

**Fig. 1:**
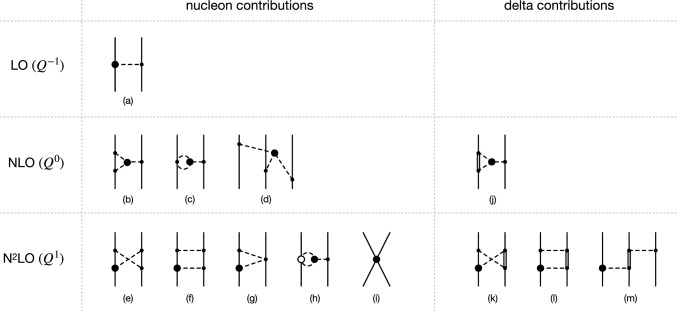
Diagrams contributing to the PVTV nuclear potentials at first three orders in the chiral EFT expansion. Solid, dashed and double lines refer to nucleons, pions and deltas, respectively. Solid dots (open circles) denote the lowest-order (subleading) PCTC vertices with $\Delta _i = 0$ ($\Delta _i = 1$). Filled circles refer to the leading PVTV pion, pion-nucleon and two-nucleon vertices with $\Delta _i = -2$, $\Delta _i = -1$ and $\Delta _i = 1$, respectively. Diagrams resulting from interchanging the order of vertices at the nucleon lines and those leading solely to renormalization of various LECs are not shown. We also do not show three-nucleon diagrams made out of the LO vertices that can be drawn at N$^2$LO but lead to vanishing contributions (in a complete analogy with the vanishing NLO PCTC three-nucleon forces)

In Fig. 1 we show various diagrams that contribute to the nuclear potentials up to N$^2$LO. Nuclear forces are defined in terms of irreducible contributions to the scattering amplitude, which cannot be generated through iterations of the Lippmann-Schwinger equation [21]. Their derivation from the effective chiral Lagrangian can be achieved using a variety of methods including time-ordered perturbation theory [7, 11, 21], S-matrix matching [12, 27], the method of unitary transformation [22, 28] and the path integral approach [29], see Refs. [30, 31] for a discussion of various approaches. On the other hand, none of the diagrams shown in Fig. 1 except the box graph (f) involve reducible time-ordered topologies, and their contributions to the nuclear forces can therefore be obtained by calculating the corresponding Feynman diagrams. At the accuracy level of our calculation, all methods mentioned above lead to identical results, provided one sticks to energy-independent nuclear potentials. The purely nucleonic diagrams (a)–(i) have been calculated in Ref. [7]. Notice that differently to the counting scheme of that study, no relativistic corrections contribute to the PVTV nuclear interactions up to N$^2$LO in our power counting scheme. We further remind the reader that the leading relativistic corrections to the one-pion exchange potential in the energy-independent formulation are suppressed by the factor of $M_\pi ^2/m_N^{2} \sim Q^4$ relative to the static result. Last but not least, there are numerous diagrams at N$^2$LO that result from one-loop dressing of the PVTV and PCTC one-pion-exchange and the leading-order (LO) PCTC 2N contact interaction, which are not shown in Fig. 1. The latter type of diagrams involve the PVTV $\pi N$-vertices and lead to vanishing N$^2$LO contributions due to the integrands being odd functions of momenta. This also applies to graphs resulting from dressing the PCTC one-pion exchange with PVTV nucleon-self-energy-type of diagrams. The remaining one-loop corrections to the one-pion exchange, except for diagrams involving the $\Delta _3$-vertex to be discussed below, only lead to renormalization of various LECs. At the order we are working, their contributions are accounted for by replacing *M*, *F*, $\mathring{g}_A$ and $g_i$ with the corresponding renormalized values $M_\pi$, $F_\pi$, ${g}_A$ and $\bar{g}_i$.

Up to the order we are working, the isospin-spin-momentum structure of the two-nucleon PVTV one- and two-pion exchange potentials can be expressed as

5 $$\begin{aligned}  &   V (\textbf{q} \, ) = \big [ V_-^\textrm{I} + W_-^\textrm{I} {\varvec{\tau }}_1 \cdot {\varvec{\tau }}_2 + V_-^\textrm{II} \tau _1^3 \tau _2^3 + V_-^\textrm{III} (\tau _1^3 + \tau _2^3) \big ] \,\nonumber \\  &   \qquad \qquad \times i (\textbf{q} \cdot \mathbf {\sigma }_1 - \textbf{q} \cdot \mathbf {\sigma }_2 ) + V_+^\textrm{IV} (\tau _1^3 - \tau _2^3) \, i (\textbf{q} \cdot \mathbf {\sigma }_1 + \textbf{q} \cdot \mathbf {\sigma }_2 ) ,\nonumber \\ \end{aligned}$$

where $\mathbf {\sigma }_i$ (${\varvec{\tau }}_i$) denote the spin (isospin) matrices of the nucleon *i*. Further, $V_-^\textrm{I} (q)$, $W_-^\textrm{I} (q)$, $V_-^\textrm{II} (q)$, $V_-^\textrm{III} (q)$ and $V_+^\textrm{IV} (q)$ are real scalar functions while $\textbf{q} = \textbf{p} \, ' - \textbf{p}$ is the momentum transfer with $\textbf{p}$ and $\textbf{p} \, '$ referring to the initial and final momenta in the center-of-mass frame and $q \equiv | \textbf{q} \, |$. The superscripts of the scalar functions refer to the usual classification scheme of the isospin dependence of the 2N force. Specifically, class-I, II, III and IV potentials correspond to isospin-invariant, charge-independence breaking, charge-symmetry breaking and isospin mixing interactions, respectively [32, 33].

The leading-order PVTV 2N potential is generated by the one-pion exchange from diagram (a) in Fig. 1, yielding [2, 4, 5, 7]

6 $$\begin{aligned} W_{-, \; 1\pi }^{\textrm{I} \; (Q^{-1})}= &   \frac{g_A \bar{g}_0}{2 F_\pi } \frac{1}{q^2 + M_\pi ^2}, \nonumber \\ V_{-, \; 1\pi }^{\textrm{II} \; (Q^{-1})}= &   \frac{g_A \bar{g}_2}{2 F_\pi } \frac{1}{q^2 + M_\pi ^2},\nonumber \\ V_{-, \; 1\pi }^{\textrm{III} \; (Q^{-1})}= &   V_{+, \; 1\pi }^{\textrm{IV} \; (Q^{-1})} \; = \; \frac{g_A \bar{g}_1}{4 F_\pi } \frac{1}{q^2 + M_\pi ^2}. \end{aligned}$$

The first corrections to the PVTV potential emerge at order $Q^0$ from one-loop diagrams (b) and (c) in Fig. 1. The latter graph yields vanishing contribution, while the long-range potential generated by diagram (b) has the form [2, 4, 7]

7 $$\begin{aligned} V_{-, \; 1\pi + 2 \pi }^{\textrm{III} \, (Q^{0})}= &   V_{+, \; 1\pi + 2 \pi }^{\textrm{IV} \, (Q^{0})} \nonumber \\= &   \!-\! \frac{5 g_A^3 \Delta _3 m_N}{64 \pi F_\pi ^3 (q ^2 \!\!+\!\! M_\pi ^2)} \big [ ( q^2 + 2 M_\pi ^2 ) A(q) \!\!+\!\! M_\pi \big ] ,\nonumber \\ \end{aligned}$$

with the loop function *A*(*q*) given by

8 $$\begin{aligned} A(q) = \frac{1}{2 q} \arctan \bigg ( \frac{q}{2 M_\pi } \bigg )\,. \end{aligned}$$

Here and in what follows, the ultraviolet divergent loop contributions to the PVTV potentials are regularized using dimensional regularization. Clearly, cutoff regularization would lead to the same result up to contributions that vanish in the infinite-cutoff limit. The expression in Eq. (7) possesses a pion pole and therefore corresponds to both one- and two-pion exchange. As done in the parity conserving sector, we employ the on-shell renormalization scheme for PVTV pion-nucleon coupling constants $\bar{g}_{1,2,3}$ by defining their renormalized values from the residues of the corresponding form factors. This way, the pion-pole contribution in Eq. (7) gets absorbed into a (finite) renormalization of the LEC $\bar{g}_1$, yielding the two-pion exchange potential

9 $$\begin{aligned} V_{-, \; 2 \pi }^{\textrm{III} \, (Q^{0})}= &   V_{+, \; 2 \pi }^{\textrm{IV} \, (Q^{0})}\nonumber \\= &   - \frac{5 g_A^3 \Delta _3 m_N}{64 \pi F_\pi ^3 (q ^2 + M_\pi ^2)} \bigg [( q^2 + 2 M_\pi ^2 ) A(q) \nonumber \\  &   - \frac{M_\pi }{2} \textrm{arctanh} \bigg ( \frac{1}{2} \bigg ) \bigg ] . \end{aligned}$$

This expression is obtained from Eq. (7) by subtracting $(q^2 + M_\pi ^2)^{-1}$ times the residue of these potentials at $q^2 = - M_\pi ^2$,

10 $$\begin{aligned}  &   \underset{q^2 = - M_\pi ^2}{Res}\ V_{-, \; 2 \pi }^{{III} \, (Q^{0})} \; = \; \underset{q^2 = - M_\pi ^2}{Res}\ V_{+, \; 2 \pi }^{{IV} \, (Q^{0})} \; \nonumber \\  &   \quad = \; - \frac{5 g_A^3 \Delta _3 m_N}{64 \pi F_\pi ^3} \bigg [ \frac{M_\pi }{2} \textrm{arctanh} \bigg ( \frac{1}{2} \bigg ) + M_\pi \bigg ]. \end{aligned}$$

The only remaining NLO contribution in the delta-less formulation of chiral EFT emerges from diagram (d) in Fig. 1. The corresponding three-nucleon force has the form [2, 4, 7]

11 $$\begin{aligned}  &   V_\textrm{3N}^{(Q^{0})}=-\frac{ig_A^3\Delta _3m_N}{4 F_\pi ^3} \frac{(\mathbf {\sigma }_1\cdot \textbf{q}_1)(\mathbf {\sigma }_2\cdot \textbf{q} _2)(\mathbf {\sigma }_3 \cdot \textbf{q}_3)}{(q_1^2+M_\pi ^2)(q_2^2+M_\pi ^2)(q_3^2+M_\pi ^2)} \nonumber \\  &   \qquad \qquad \times \big [({\varvec{\tau }}_2\cdot {\varvec{\tau }}_3)\tau _1^3+({\varvec{\tau }}_1\cdot {\varvec{\tau }}_3)\tau _2^3+({\varvec{\tau }}_1\cdot {\varvec{\tau }}_2)\tau _3^3 \big ] , \end{aligned}$$

where $\textbf{q}_i = \textbf{p}_i \,' - \textbf{p}_i$ is the momentum transfer of the nucleon *i*, and $\textbf{q}_1 + \textbf{q}_2 + \textbf{q}_3 = 0$ because of momentum conservation.

Next, at N$^2$LO, the contributions to the PVTV 2N potential from diagrams (e), (f), (g) and (h) in Fig. 1 have the form

12 $$\begin{aligned} W_{-, \; 2 \pi }^{\textrm{I} \, (Q^{1})}= &   - \frac{g_A (2 \bar{g}_0 +\bar{g}_2)}{32 \pi ^2 F_\pi ^3 (q^2 + 4 M_\pi ^2)} \nonumber \\  &   \times \big [ (8 g_A^2 -4) M_\pi ^2 + (3 g_A^2 -1 ) q^2 \big ] L(q),\nonumber \\ V_{-, \; 2 \pi }^{\textrm{II} \, (Q^{1})}= &   \frac{g_A \bar{g}_2}{32 \pi ^2 F_\pi ^3 (q^2 + 4 M_\pi ^2)} \nonumber \\  &   \times \big [ (8 g_A^2 -4) M_\pi ^2 + (3 g_A^2 -1 ) q^2 \big ] L(q),\nonumber \\ V_{-, \; 1\pi +2 \pi }^{\textrm{III} \, (Q^{1})}= &   V_{+, \; 1\pi +2 \pi }^{\textrm{IV} \, (Q^{1})} = \frac{5 g_A \Delta _3 m_N}{192 \pi ^2 F_\pi ^3} \nonumber \\  &   \times \bigg [\frac{3 M_\pi ^2}{q^2 + M_\pi ^2} (8 c_1 - c_2 - 2 c_3 ) - (c_2 + 6 c_3 ) \bigg ] L (q),\nonumber \\ \end{aligned}$$

where only terms nonpolynomial in momenta are listed. The loop function *L*(*q*) is given by

13 $$\begin{aligned} L(q) = \frac{s}{q} \ln \bigg ( \frac{s+q}{2 M_\pi } \bigg )\,, \end{aligned}$$

with $s = \sqrt{q^2 + 4 M_\pi ^2}$. The $\bar{g}_0$-contribution to the potential $W_{-, \; 2 \pi }^{\textrm{I} \, (Q^{1})}$ agrees with the one given in Ref. [7]. On the other hand, the $\bar{g}_2$-contribution to $W_{-, \; 2 \pi }^{\textrm{I} \, (Q^{1})}$ and the potential $V_{-, \; 2 \pi }^{\textrm{II} \, (Q^{1})}$ differ from the corresponding expressions in Ref. [7] by the factors of 3/4 and 1/2, respectively. These discrepancies originate from an incorrect factor in front of the second term in Eq. (B16) of Ref. [7] obtained by adding the expressions in Eqs. (B14) and (B15). As for $V_{-, \; 1\pi +2 \pi }^{\textrm{III} \, (Q^{1})}$ and $V_{-, \; 1\pi +2 \pi }^{\textrm{IV} \, (Q^{1})}$, the term $\propto c_1$ agrees with the one given in Ref. [7], while those $\propto c_2$ and $\propto c_3$ differ from the expressions given in that paper. Up to equation (B28) of Ref. [7], we actually found the same expressions as given in that paper. The discrepancy seems to originate from evaluating the integral in front of $c_2+c_3$ in Eq. (B28) of that work. The ultraviolet divergences generated by loop integrals are absorbed into renormalization of $\bar{g}_1$ and of five N$^2$LO PVTV contact interactions, whose explicit form can be found in Ref. [7]. Similarly to the NLO potentials in Eq. (7), the pion-pole contribution to $V_{-, \; 1\pi +2 \pi }^{\textrm{III} \, (Q^{1})}$ and $V_{-, \; 1\pi +2 \pi }^{\textrm{IV} \, (Q^{1})}$ in Eq. (12) gets absorbed into renormalization of the LEC $\bar{g}_1$, leading to the two-pion exchange potential

14 $$\begin{aligned}  &   V_{-, \; 2 \pi }^{\textrm{III} \, (Q^{1})} = V_{+, \; 2 \pi }^{\textrm{IV} \, (Q^{1})} = \frac{5 g_A \Delta _3 m_N}{192 \pi ^2 F_\pi ^3}\nonumber \\  &   \quad \times \bigg [\frac{3 M_\pi ^2}{q^2 + M_\pi ^2} (8 c_1 - c_2 - 2 c_3 ) \bigg (L(q) - \frac{\pi }{2 \sqrt{3}} \bigg )\nonumber \\  &   \qquad - (c_2 + 6 c_3 )L (q) \bigg ]. \end{aligned}$$

We now turn to the delta-isobar contributions. The NLO contribution is generated by diagram (j) in Fig. 1 and can be expressed as

15 $$\begin{aligned}  &   V_{-, \; 1\pi +2 \pi , \; \Delta }^{\textrm{III}\, (Q^{0})} =V_{+, \; 1\pi +2 \pi , \; \Delta }^{\textrm{IV}\, (Q^{0})} = -\frac{5\Delta _3 g_A h_A^2 m_N}{36 \pi ^2 F_\pi ^3}\nonumber \\  &   \ \ \frac{\Delta }{M_\pi ^2+q^2}\left[ (2M_\pi ^2+q^2-2\Delta ^2)D(q)-L(q)\right] , \end{aligned}$$

where $\Delta$ denotes the mass splitting $\Delta = m_\Delta - m_N$ and we have introduced the loop function

16 $$\begin{aligned} D(q)=\frac{1}{\Delta }\int _{2M_\pi }^\infty d\mu \frac{1}{\mu ^2+q^2}\arctan \left( \frac{\sqrt{\mu ^2-4M_\pi }}{2\Delta }\right) \,. \end{aligned}$$

Subtracting out the pion-pole contribution that renormalizes the LEC $\bar{g}_1$, we obtain the corresponding two-pion exchange potential in the form

17 $$\begin{aligned}  &   V_{-, \; 2 \pi , \; \Delta }^{\textrm{III}\, (Q^{0})} =V_{+, \; 2 \pi , \; \Delta }^{\textrm{IV}\, (Q^{0})}= -\frac{5\Delta _3 g_A h_A^2 m_N}{36 \pi ^2 F_\pi ^3}\frac{\Delta }{M_\pi ^2+q^2}\nonumber \\  &   \quad \times \left[ (2M_\pi ^2+q^2-2\Delta ^2)D(q)\right. \nonumber \\  &   \qquad \left. -(M_\pi ^2 - 2 \Delta ^2) D(i M_\pi ) - L(q) + \frac{\pi }{2\sqrt{3}}\right] . \end{aligned}$$

At N$^2$LO, we find for the 2N-diagrams (k) and (l) in Fig. 1

18 $$\begin{aligned} V_{-, \; 2 \pi , \; \Delta }^{\textrm{I}\, (Q^{1})}= &   -\frac{g_A h_A^2(3\bar{g}_0+\bar{g}_2)}{18 \pi F_\pi ^3 \Delta }(2 M_\pi ^2+q^2) A(q),\nonumber \\ W_{-, \; 2 \pi , \; \Delta }^{\textrm{I}\, (Q^{1})}= &   - \frac{(2\bar{g}_0+\bar{g}_2)g_A h_A^2}{36\pi ^2F_\pi ^3}\nonumber \\  &   \quad \times \left[ (2M_\pi ^2+q^2-2\Delta ^2)D(q)-L(q)\right] ,\nonumber \\ V_{-, \; 2 \pi , \; \Delta }^{\textrm{II}\, (Q^{1})}= &   \frac{\bar{g}_2g_A h_A^2}{36\pi ^2 F_\pi ^3}\left[ (2M_\pi ^2+q^2-2\Delta ^2)D(q) -L(q) \right] ,\nonumber \\ V_{-, \; 2 \pi , \; \Delta }^{\textrm{III}\, (Q^{1})}= &   V_{+, \; 2 \pi , \; \Delta }^{\textrm{IV}\, (Q^{1})} = -\frac{g_A h_A^2 \bar{g}_1}{36 \pi F_\pi ^3\Delta } (2M_\pi ^2+q^2) A(q).\nonumber \\ \end{aligned}$$

Finally, the diagram (m) generates the 3N-potential given by

19 $$\begin{aligned}  &   V_{3N}^{(Q^{1})} = \frac{4g_A h_A^2}{9F_\pi ^3\Delta } \frac{\textbf{q}_1\cdot \textbf{q}_2}{ (q_1^2+M_\pi ^2)(q_3^2+M_\pi ^2)} \nonumber \\  &   \qquad \qquad \times \bigg \{ \frac{\bar{g}_1}{2}\Big [\big (\mathbf {\sigma }_1\cdot \textbf{q}_1-\mathbf {\sigma }_3\cdot \textbf{q}_3 \big ) \big (\tau _1^3+\tau _3^3\big )\nonumber \\  &   \qquad \qquad +\big (\mathbf {\sigma }_1\cdot \textbf{q}_1+\mathbf {\sigma }_3\cdot \textbf{q}_3\big ) \big (\tau _1^3-\tau _3^3\big )\Big ] \nonumber \\  &   \qquad + \bar{g}_2 \, \tau _1^3\tau _3^3 \, \big (\mathbf {\sigma }_1\cdot \textbf{q}_1- \mathbf {\sigma }_3\cdot \textbf{q}_3 \big ) \bigg \} \; +\; \mathrm {5 \; permutations}. \nonumber \\ \end{aligned}$$

As a consistency check of the calculated $\Delta$ contributions to the PVTV 2N potential, we have looked at their representation in terms of resonance saturation of LECs in the delta-less approach. Using the $1/\Delta$ expansion of the loop function *D*(*q*),

20 $$\begin{aligned} D(q)=-\frac{1}{2\Delta ^2}L(q)-\frac{1}{24\Delta ^4}(4M_\pi ^2+q^2)L(q) +\mathcal {O}(\Delta ^{-6}), \end{aligned}$$

one observes that the $\mathcal {O} (\Delta ^{-1})$ contributions stemming from the $1/\Delta$ expansion of $V_{-, \; 2 \pi , \; \Delta }^{\textrm{III}\,({Q^0})}$ and $V_{-, \; 2 \pi , \; \Delta }^{\textrm{IV}\,({Q^0})}$ in Eq. (17) are reproduced by setting the LECs $c_i$ in Eq. (14) to their $\Delta$-resonance-saturation values [34]

21 $$\begin{aligned} c_1=0,\quad c_2=-c_3=\frac{4h_A^2}{9\Delta }\,. \end{aligned}$$

To verify the consistency of the N$^2$LO-$\Delta$ contributions given in Eq. (18), we have worked out the triangle diagram of type (g) in Fig. 1, where the Weinberg-Tomozawa vertex is replaced by the subleading $\pi \pi NN$ vertex proportional to the LECs $c_i$. This yields the N$^3$LO contributions of the form

22 $$\begin{aligned}  &   V_{-,\; 2 \pi }^{\textrm{I}\,({Q^2})} = \frac{g_A(3\bar{g}_0+\bar{g}_2)}{8\pi F_\pi ^3}\big [M_\pi ^2(4c_1-2c_3)-q^2c_3 \big ]A(q), \nonumber \\  &   V_{-,\; 2 \pi }^{\textrm{III}\,({Q^2})} = V_{+,\; 2 \pi }^{\textrm{IV}\,({Q^2})} = \frac{g_A\bar{g}_1}{16\pi F_\pi ^3}\big [M_\pi ^2(4c_1-2c_3)-q^2c_3\big ]A(q). \nonumber \\ \end{aligned}$$

Again, it is easy to see that these expressions coincide with the $\mathcal {O} (\Delta ^{-1})$ terms resulting from the $1/\Delta$-expansion of Eq. (18) when using the values of $c_i$’s specified in Eq. (21). We have also verified the resonance-saturation picture for the three-nucleon force given in Eq. (19).

## Large-distance behavior of the PVTV 2N potentials

The strength of the calculated long-range potentials can be read off from the corresponding coordinate-space expressions. Following the notation in Eq. (5), we define the profile functions $\tilde{V}_-^\textrm{I} (r)$, $\tilde{W}_-^\textrm{I} (r)$, $\tilde{V}_-^\textrm{II} (r)$, $\tilde{V}_-^\textrm{III} (r)$ and $\tilde{V}_+^\textrm{IV} (r)$ via

23 $$\begin{aligned}  &   \tilde{V} (\textbf{r} \, ) = \big [ \tilde{V}_-^\textrm{I} + \tilde{W}_-^\textrm{I} {\varvec{\tau }}_1 \cdot {\varvec{\tau }}_2 + \tilde{V}_-^\textrm{II} \tau _1^3 \tau _2^3 + \tilde{V}_-^\textrm{III} (\tau _1^3 + \tau _2^3) \big ] \, \nonumber \\  &   \qquad \qquad \times (\hat{r} \cdot \mathbf {\sigma }_1 - \hat{r} \cdot \mathbf {\sigma }_2 ) + \tilde{V}_+^\textrm{IV} (\tau _1^3 - \tau _2^3) \, (\hat{r} \cdot \mathbf {\sigma }_1 + \hat{r} \cdot \mathbf {\sigma }_2 ) ,\nonumber \\ \end{aligned}$$

where $\textbf{r}$ is the relative distance between the nucleons, $r \equiv | \textbf{r} \,|$ and $\hat{r} \equiv \textbf{r} / r$. The PVTV one-pion exchange potential has the form

24 $$\begin{aligned} \tilde{W}_{-, \; 1\pi }^{\textrm{I}\,({Q^{-1}})} (r)= &   - \frac{g_A \bar{g}_0}{8 \pi F_\pi } \frac{e^{-x}}{r^2} (1 + x), \nonumber \\ \tilde{V}_{-, \; 1\pi }^{\textrm{II}\,({Q^{-1}})} (r)= &   - \frac{g_A \bar{g}_2}{8 \pi F_\pi } \frac{e^{-x}}{r^2} (1 + x),\nonumber \\ \tilde{V}_{-, \; 1\pi }^{\textrm{III} \; (Q^{-1})} (r)= &   \tilde{V}_{+, \; 1\pi }^{\textrm{IV} \; (Q^{-1})} (r) \; = \; - \frac{g_A \bar{g}_1}{16 \pi F_\pi } \frac{e^{-x}}{r^2} (1 + x),\nonumber \\ \end{aligned}$$

where we have introduced a dimensionless variable $x = M_\pi r$.

**Table 1:** Low-energy constants accompanying various PVTV vertices that govern the long-range contributions to the PVTV 2N force up to N$^2$LO in the chiral EFT expansion. NLO and N$^2$LO refer to the two-pion exchange contributions in the delta-less formulation, which are generated by diagrams (b), (c) and (e)–(h) of Fig. 1. NLO-$\Delta$ and N$^2$LO-$\Delta$ refer to the additional contributions in the delta-full framework stemming from diagrams (j), (k) and (l)

	LO ($V_{1 \pi }$)	NLO ($V_{2 \pi }$)	N$^2$LO ($V_{2 \pi }$)	NLO-$\Delta$ ($V_{2 \pi }$)	N$^2$LO-$\Delta$ ($V_{2 \pi }$)
$V_-^\textrm{I}$	–	–	–	–	$3 \bar{g}_0 + \bar{g}_2$
$W_-^\textrm{I}$	$\bar{g}_0$	–	$2 \bar{g}_0 + \bar{g}_2$	–	$2 \bar{g}_0 + \bar{g}_2$
$V_-^\textrm{II}$	$\bar{g}_2$	–	$\bar{g}_2$	–	$\bar{g}_2$
$V_-^\textrm{III}$	$\bar{g}_1$	$\Delta _3$	$\Delta _3$	$\Delta _3$	$\bar{g}_1$

The Fourier transform of the two-pion exchange contributions can be facilitated using their spectral representation [27], which yields the large-distance behavior

25 $$\begin{aligned} \tilde{X}_\pm (r) =-\frac{1}{2\pi ^2r^2}\int _{2M_\pi }^{\infty } d\mu \mu e^{-\mu r}\, (1+\mu r) \, \textrm{Im} X_\pm (-i\mu )\,, \end{aligned}$$

in terms of the spectral functions $\textrm{Im} X_\pm (-i\mu )$ with *X* staying for *V* or *W*. Hence, to calculate the coordinate expressions, we need the imaginary parts of the loop functions. By analytic continuation one obtains

26 $$\begin{aligned} \textrm{Im} L(-i\mu )= &   -\frac{\pi }{2} \frac{\sqrt{\mu ^2-4M_\pi ^2}}{\mu },\nonumber \\ \textrm{Im} A(-i\mu )= &   \frac{\pi }{4\mu },\nonumber \\ \textrm{Im} D(-i\mu )= &   \frac{\pi }{2\Delta \mu } \arctan {\frac{\sqrt{\mu ^2-4M_\pi ^2}}{2\Delta }}. \end{aligned}$$

Notice that the coordinate-space expressions given below become meaningless at short distances of *r* well below $M_\pi ^{-1}$, which is outside of the convergence range of the chiral expansion. When calculating nuclear observables by solving the Schrödinger equation, this unphysical short-range behavior is removed by a regulator.

For the leading two-pion exchange contributions given in Eq. (9), we obtain

27 $$\begin{aligned} \tilde{V}_{-, \; 2 \pi }^{\textrm{III} \, (Q^{0})} (r)&=\tilde{V}_{+, \; 2 \pi }^{\textrm{IV} \, (Q^{0})} (r) \; \nonumber \\&= \; \frac{5 g_A^3 \Delta _3 m_N}{1024 \pi ^2 F_\pi ^3} \frac{1}{r^3} \nonumber \\&\quad \ \ \times \Big [ x (x-1) e^x \textrm{Ei} (-3x) + 4(1+x) e^{-2x}\nonumber \\&\quad \ + x(1+x) e^{-x} \textrm{Ei} (-x) \Big ], \end{aligned}$$

where $\textrm{Ei} (z)$ is the exponential integral function given by $\textrm{Ei} (z) = - \int _{z}^\infty e^{-t}/t dt$. For the subleading two-pion exchange contributions specified in Eq. (12), we have only succeeded to obtain analytical results for $\tilde{W}_{-, \; 2 \pi }^{\textrm{I} \, (Q^{1})} (r)$ and $\tilde{V}_{-, \; 2 \pi }^{\textrm{II} \, (Q^{0})} (r)$:

28 $$\begin{aligned} \tilde{W}_{-,\;2\pi }^{\textrm{I}\,(Q^{1})}&=-\frac{(2\bar{g}_0+\bar{g}_2)g_AM_\pi }{64\pi ^3F_\pi ^3 r^3}\nonumber \\&\quad \ \times \Big \{2x(4g_A^2-1)K_0(2x)+\big [g_A^2(9+4x^2)-3\big ]K_1(2x)\Big \} , \nonumber \\ \tilde{V}_{-,\;2\pi }^{\textrm{II}\,(Q^{1})}&= \frac{\bar{g}_2g_AM_\pi }{64\pi ^3F_\pi ^3r^3}\Big \{2x(4g_A^2-1) K_0(2x)\nonumber \\&\quad \ +\big [g_A^2(9+4x^2)-3\big ] K_1(2x)\Big \}, \end{aligned}$$

where $K_n (z)$ refer to the modified Bessel functions of the second kind. The remaining potentials $\tilde{V}_{-,\;2\pi }^{\textrm{III}\,(Q^{1})} (r)$ and $\tilde{V}_{+,\;2\pi }^{\textrm{IV}\,(Q^{1})} (r)$ can be obtained numerically by performing the spectral integral in Eq. (25) using Eqs. (12) and (26).

For the two-pion exchange potentials mediated by the intermediate delta excitations, compact analytical expressions are only available for $\tilde{V}_{-,\;2\pi , \; \Delta }^{\textrm{I}\,(Q^{1})} (r)$, $\tilde{V}_{-,\;2\pi , \; \Delta }^{\textrm{III}\,(Q^{1})} (r)$ and $\tilde{V}_{+,\;2\pi , \; \Delta }^{\textrm{IV}\,(Q^{1})} (r)$,

29 $$\begin{aligned} \tilde{V}_{-, \; 2 \pi , \; \Delta }^{\textrm{I}\, (Q^{1})} (r)= &   -\frac{(3\bar{g}_0+\bar{g}_2)g_A h_A^2}{36 \pi ^2 F_\pi ^3\Delta }\frac{e^{-2x}}{r^5}\nonumber \\  &   \quad (1+x)\big (2+x(2+x)\big ), \end{aligned}$$

30 $$\begin{aligned} \tilde{V}_{-, \; 2 \pi , \; \Delta }^{\textrm{III}\, (Q^{1})} (r)= &   \tilde{V}_{+, \; 2 \pi , \; \Delta }^{\textrm{IV}\, (Q^{1})} (r)\nonumber \\  &   = -\frac{\bar{g}_1g_A h_A^2}{72\pi ^2 F_\pi ^3 \Delta }\frac{e^{-2x}}{r^5}(1+x)\big (2+x(2+x)\big ),\nonumber \\ \end{aligned}$$

while the remaining potentials have to be calculated numerically using Eqs. (17), (18), (25) and (26).

We are now in the position to present numerical results and discuss the impact of treating the delta isobar as an explicit degree of freedom on the PVTV 2N potentials. Here and in what follows, we use the following values for the various constants: $M_\pi =138$ MeV, $F_\pi = 92.4$ MeV, $m_N = 939$ MeV, $\Delta = 293$ MeV. Further, we use the effective value for the axial coupling constant of the nucleon, $g_A = 1.29$, which takes into account the Goldberger-Treiman discrepancy. For the pion-nucleon-delta coupling constant $h_A$, we adopt the value of $h_A = 1.34$ fixed from the width of the delta resonance. For the LECs $c_i$, we employ the values $c_1=-0.74$ GeV$^{-1}$, $c_2=1.81$ GeV$^{-1}$ and $c_3=-3.61$ GeV$^{-1}$ taken from the order-$Q^2$ heavy-baryon-NN fit of Ref. [35]. When performing the calculations in the formulation with explicit delta isobars, we subtract from these values the delta contributions specified in Eq. (21) and use $c_1=-0.74$ GeV$^{-1}$, $c_2=-0.91$ GeV$^{-1}$ and $c_3=-0.89$ GeV$^{-1}$. This leaves us with the only unknown (dimensionless) LECs $\bar{g}_0$, $\bar{g}_1$, $\bar{g}_2$ and $\Delta _3$ associated with the PVTV vertices. The dependence of the obtained contributions to the one- and two-pion exchange 2N potentials on these PVTV LECs is summarized in Table 1.

Notice that while the NLO-$\Delta$ contribution to the two-pion exchange is implicitly taken into account in the delta-less framework through resonance saturation of the LECs $c_i$ entering the N$^2$LO potential, the N$^2$LO-$\Delta$ terms go beyond the N$^2$LO accuracy level of the delta-less formulation of chiral EFT. It is thus interesting to look at the strength of the resulting potentials, which may give hints about their phenomenological importance when analyzing PVTV signals in nuclear systems.

**Fig. 2:**
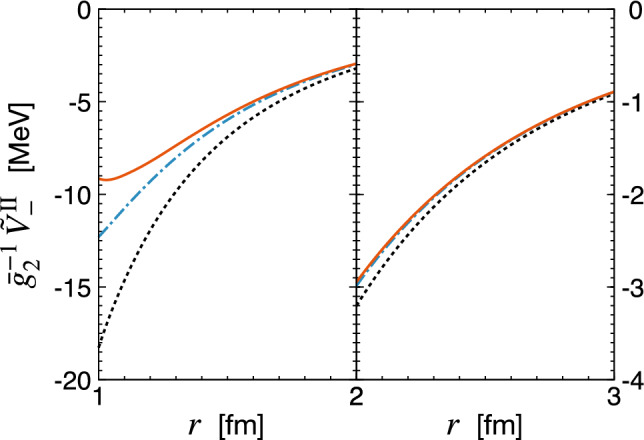
Chiral EFT expansion of the PVTV 2N potential $\tilde{V}_{-}^{\textrm{II}} (r)$ as a function of the distance *r* between the nucleons. Black dotted lines show the LO result due to the one-pion exchange, while blue dashed-dotted and red solid lines refer to the complete N$^2$LO results in the delta-less and delta-full formulations of chiral EFT, respectively. Notice that there are no NLO contributions to $\tilde{V}_{-}^{\textrm{II}} (r)$

In Fig. 2, we compare the chiral expansion of the potential $\tilde{V}_-^\textrm{II} (r)$ in the delta-less and delta-full formulations of chiral EFT. Notice that this structure does not receive any corrections at order $Q^0$ (i.e., at NLO). Clearly, the long-range part of the interaction is completely dominated by the one-pion exchange, but the effects of the two-pion exchange become visible at distances below about 2 fm. For the case at hand, the delta-resonance contribution at N$^2$LO turns out to be smaller in magnitude than the nucleonic one at the same order and it, in fact, becomes negligibly small beyond $r \sim 1.5$ fm.

**Fig. 3:**
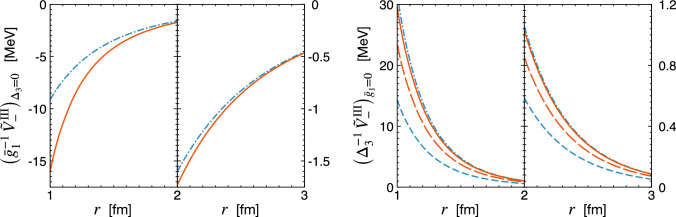
Chiral EFT expansion of the PVTV 2N potential $\tilde{V}_{-}^{\textrm{III}} (r)$ as a function of the distance *r* between the nucleons. Left and right panels show the results for the contributions proportional to the PVTV LECs $\bar{g}_1$ and $\Delta _3$, respectively. Blue short-dashed and red long-dashed lines show the complete NLO results in the delta-less and delta-full formulations of chiral EFT, respectively. For the remaining notation see Fig. 2

Next, in Fig. 3, we compare the results obtained for the PVTV potential $\tilde{V}_-^\textrm{III} (r)$ using the delta-less and delta-full formulations of chiral EFT. Since the contributions at different orders depend on two unknown PVTV LECs $\bar{g}_1$ and $\Delta _3$, we have decided to show the corresponding potentials separately. The $\bar{g}_1$-contribution is dominated by the one-pion exchange, which does not receive any corrections at NLO and N$^2$LO in the delta-less formulation. When the delta-isobar is treated as an explicit degree of freedom, the $\bar{g}_1$-part of the potential $\tilde{V}_-^\textrm{III} (r)$ receives a correction at N$^2$LO, which appears to be quite sizable at distances $r \lesssim 1.5$ fm. The rather large contribution of the delta resonance is consistent with the enhancement by a factor of $\pi$, as one can see from Eq. (18). The potential $\tilde{V}_-^\textrm{III} (r)$ receives the two-pion exchange contributions at NLO and N$^2$LO.^3^ In the delta-less framework, the N$^2$LO correction to $\tilde{V}_-^\textrm{III} (r)$ is large, being numerically comparable to the nominally dominant NLO contribution. The convergence of the chiral EFT expansion is considerably improved in the delta-full framework, where the dominant NLO-$\Delta$ result $\tilde{V}_{-, \; 2 \pi , \; \Delta }^{\textrm{III}\, (Q^{0})} (r)$ already takes into account a significant part of the N$^2$LO correction. In particular, it includes the part of the N$^2$LO potential $\tilde{V}_{-, \; 2 \pi }^{\textrm{III} \, (Q^{1})} (r)$, which is driven by the delta-resonance-saturation values of the LECs $c_i$ quoted in Eq. (21). The potential $\tilde{V}_-^\textrm{III} (r)$ does not receive any N$^2$LO contribution $\propto \Delta _3$ from the intermediate delta excitation, so that the N$^2$LO correction is given by the same expression in both the delta-less and delta-full formulations. However, in the approach with explicit delta-isobar, the LECs $c_{2,3}$ are considerably smaller in magnitude, so that the N$^2$LO-$\Delta$ correction has a more natural size. The final results at N$^2$LO, however, appear to be rather similar in both frameworks. This may be viewed as an indication that effects of the delta-isobar beyond the leading contributions governed by the resonance saturation of $c_i$’s are small.

**Fig. 4:**
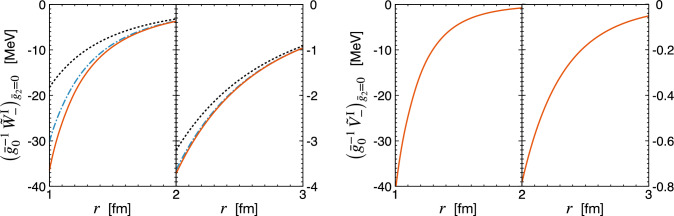
Chiral EFT expansion of the PVTV 2N potentials $\tilde{W}_{-}^{\textrm{I}} (r)$ (left panel) and $\tilde{V}_{-}^{\textrm{I}} (r)$ (right panel) as a function of the distance *r* between the nucleons. In both cases, the PVTV LEC $\bar{g}_2$ is set to zero. For notation see Figs. 2 and 3

Finally, we turn to the isospin-invariant PVTV potentials $\tilde{V}_-^\textrm{I} (r)$ and $\tilde{W}_-^\textrm{I} (r)$. As shown in Table 1, these potentials are driven by different linear combinations of the unknown PVTV coupling constants $\bar{g}_0$ and $\bar{g}_2$, which complicates the comparison of their strengths at different EFT orders. However, in most scenarios of CP violation in and beyond the Standard Model, the coupling $\bar{g}_2$ is strongly suppressed relative to $\bar{g}_0$ [2], see Table I of Ref. [10]. In particular, in the scenario with strong CP violation generated by the QCD $\theta$-term, one even has $\Delta _3, \bar{g}_1, \bar{g}_2 \ll \bar{g}_0 \sim \mathcal {O} (1)$. In the following, we therefore set $\bar{g}_2 = 0$ for the sake of comparison of the delta-full and delta-less results. Our predictions for the potentials $\tilde{W}_-^\textrm{I} (r)$ and $\tilde{V}_-^\textrm{I} (r)$ are shown in Fig. 4. The isovector potential $\tilde{W}_-^\textrm{I} (r)$ receives the contribution from the one-pion exchange at LO, which dominates its large-distance behavior. The N$^2$LO two-pion exchange potential $\tilde{W}_{-, \; 2 \pi }^{\textrm{I} \, (Q^{1})} (r)$ provides a sizable correction at $r \lesssim 2$ fm, while the corresponding delta-resonance contribution $\tilde{W}_{-, \; 2 \pi , \; \Delta }^{\textrm{I}\, (Q^{1})} (r)$ is fairly small, especially at large distances. The most interesting finding of our work is the PVTV isoscalar potential $\tilde{V}_-^\textrm{I} (r)$, which is shown in the right panel of Fig. 4. It receives no contribution from the one-pion exchange and two-pion exchange up-to-and-including N$^2$LO in the delta-less formulation. On the other hand, we find a very strong two-pion exchange contribution at N$^2$LO-$\Delta$ generated by diagrams (k) and (j) in Fig. 1 with an intermediate delta-excitation. In the delta-less framework, these contributions appear first at N$^3$LO from triangle diagrams with one insertion of the subleading pion-nucleon vertices proportional to $c_i$’s, see Eq. (22). The strong enhancement of $\tilde{V}_{-, \; 2 \pi , \; \Delta }^{\textrm{I}\, (Q^{1})} (r)$ relativ to the expectations based on the power counting is similar to what is observed for the isoscalar central PCTC two-pion exchange potential [36]. As one can see from Eq. (18) or Eq. (22), the enhancement originates from a combination of factors including the smallness of the delta-nucleon mass splitting $\Delta$, the enhancement by a factor of $\pi$ and a large numerical prefactor. As a result, the potential $\tilde{V}_{-, \; 2 \pi , \; \Delta }^{\textrm{I}\, (Q^{1})} (r)$ appears to be even stronger than all of the NLO PVTV two-pion exchange components we have calculated. Thus, one may expect from this novel contribution to significantly affect the predictions for PVTV nuclear observables. Notice, however, that the potential $\tilde{V}_{-}^{\textrm{I}\, (Q^{1})}$ also receives a contribution from a contact interaction [8], which may, at least to some extent, mimic the two-pion exchange potential generated by the intermediate delta excitation.

## Summary and conclusions

In this paper, we have derived the contributions to the long-range parity-violating time-reversal-violating nuclear forces up through N$^2$LO using the formulation of chiral EFT with explicit delta-isobar degrees of freedom. The main results of our study can be summarized as follows:

- We have discussed the PVTV effective Lagrangian for pions, nucleons and the delta-isobar and argued that PVTV $\pi N \Delta$-vertices must involve at least one derivative, thus being suppressed relative to the leading derivative-less pion and pion-nucleon vertices. Accordingly, no additional unknown coupling constants appear in the delta-contributions to the PVTV nuclear forces up to N$^2$LO. Using the delta-full formulation of chiral EFT thus allows one to resum the dominant contributions beyond N$^2$LO of the delta-less framework in a parameter-free way, which may lead to an improved convergence of the EFT expansion.
- Our renormalized expressions for the long-range two- and three-nucleon PVTV forces in the delta-less chiral EFT framework mostly agree with the ones given in the literature, but we also found some differences for the N$^2$LO two-pion exchange potentials as compared with Ref. [7].
- We have worked out the delta contributions to the long-range two- and three-nucleon potentials at NLO-$\Delta$ and N$^2$LO-$\Delta$ using the small-scale expansion scheme by counting $\Delta \sim M_\pi$. We have verified that all obtained contributions are consistent with the delta-resonance saturation picture, i.e., the dominant terms after performing the $1/\Delta$-expansion coinside with the one-order-higher delta-less expressions upon using the values of the LECs $c_i$ quoted in Eq. (21).
- By analyzing the potentials in coordinate space, we found indications of an improved convergence of the small-scale expansion compared to the delta-less formulation of chiral EFT for the PVTV potential $\tilde{V}_-^\textrm{III} (r)$. As the most interesting result of this study, we found a strong isospin-invariant PVTV two-pion exchange potential $\tilde{V}_-^\textrm{I} (r)$ driven by the intermediate delta excitation, which is completely missing in the delta-less framework at the N$^2$LO accuracy level.

The analytical expressions for various potentials we obtained are well suited for applications to few- and many-nucleon systems upon introducing an appropriate regulator as done e.g. in Ref. [37]. It would be interesting to see phenomenological implications of our findings on the relationships between the PVTV couplings $\Delta _3$, $\bar{g}_i$ and PVTV few-nucleon observables such as, e.g., the electric dipole moments of light nuclei, see Ref. [10] for a review.

## Data Availability

My manuscript has no associated data. [Author’s comment: No datasets have been generated during and/or analysed during the current study.]

## Footnotes

1 Here and in what follows, the term irreducible refers to time-ordered diagrams that do not involve purely nucleonic intermediate states. Such diagrams are free of infrared divergences in the $m_N \rightarrow \infty$ limit, and their contributions obey the standard power counting of chiral perturbation theory [21].

2 In our counting scheme, it would be more appropriate to express this LEC in terms of the dimensionless coupling $\Delta _3$ as $\Delta _3 \Lambda _b$, but we decided to keep the established notation.

3 As explained in Sect. 2, diagrams (b) and (j) of Fig. 1 also generate contributions to the one-pion exchange potential which, however, only lead to renormalization of the LEC $\bar{g}_1$ and are thus not shown in the right panel of Fig. 3.

## Code Availability Statement

My manuscript has no associated code/software. [Author’s comment: Code/Software sharing not applicable to this article as no code/software was generated or analysed during the current study.]
